# Intraoral Scanner Technologies: A Review to Make a Successful Impression

**DOI:** 10.1155/2017/8427595

**Published:** 2017-09-05

**Authors:** Raphaël Richert, Alexis Goujat, Laurent Venet, Gilbert Viguie, Stéphane Viennot, Philip Robinson, Jean-Christophe Farges, Michel Fages, Maxime Ducret

**Affiliations:** ^1^Faculté d'Odontologie, Université Lyon 1, Université de Lyon, Lyon, France; ^2^Service de Consultations et Traitements Dentaires, Hospices Civils de Lyon, Lyon, France; ^3^Laboratoire P2S, Parcours Santé Systémique, Université Lyon 1, Université de Lyon, Lyon, France; ^4^DRCI, Hospices Civils de Lyon, Lyon, France; ^5^Laboratoire de Biologie Tissulaire et Ingénierie Thérapeutique, UMR5305 CNRS/Université Lyon 1, UMS3444 BioSciences Gerland, Lyon Sud, Lyon, France; ^6^Laboratoire de Bioingénierie et Nanosciences, EA 4203, UFR d'Odontologie, Université Montpellier 1, Montpellier, France

## Abstract

To overcome difficulties associated with conventional techniques, impressions with IOS (intraoral scanner) and CAD/CAM (computer-aided design and manufacturing) technologies were developed for dental practice. The last decade has seen an increasing number of optical IOS devices, and these are based on different technologies; the choice of which may impact on clinical use. To allow informed choice before purchasing or renewing an IOS, this article summarizes first the technologies currently used (light projection, distance object determination, and reconstruction). In the second section, the clinical considerations of each strategy such as handling, learning curve, powdering, scanning paths, tracking, and mesh quality are discussed. The last section is dedicated to the accuracy of files and of the intermaxillary relationship registered with IOS as the rendering of files in the graphical user interface is often misleading. This overview leads to the conclusion that the current IOS is adapted for a common practice, although differences exist between the technologies employed. An important aspect highlighted in this review is the reduction in the volume of hardware which has led to an increase in the importance of software-based technologies.

## 1. Introduction

Since the eighteenth century, conventional impression techniques have been used to register the three-dimensional geometry of dental tissues. Nevertheless, volumetric changes of impression materials and expansion of dental stone seem error-prone, and thus the process requires the services of an excellent dental laboratory [1–3]. To overcome these difficulties, impression with IOS (intraoral scanner) was developed for dental practice [4]. The implementation of the IOS device in dental practices coincided with the development of CAD/CAM (computer-aided design and manufacturing) technology in dentistry, with numerous advantages for practitioners. Nowadays, IOS and CAD/CAM provide easier planning of treatment, case acceptance, communication with laboratories, reduced operative time, storage requirements, and reduced treatment times [5–7]. The last decade has seen an increasing number of optical IOS, and these are based on different technologies; the choice of which may impact on clinical use [6].

To allow the practitioner to make an informed choice before purchasing or renewing an IOS, this article is divided in three distinct parts. The first presents the different technologies employed by the current IOS for the capture of image and the generation of a digital file by the software, the second is dedicated to the clinical pitfalls associated with these technologies during IOS use, and the last part reports on the accuracy of these current technologies.

## 2. IOS Technologies

**Figure 1:**
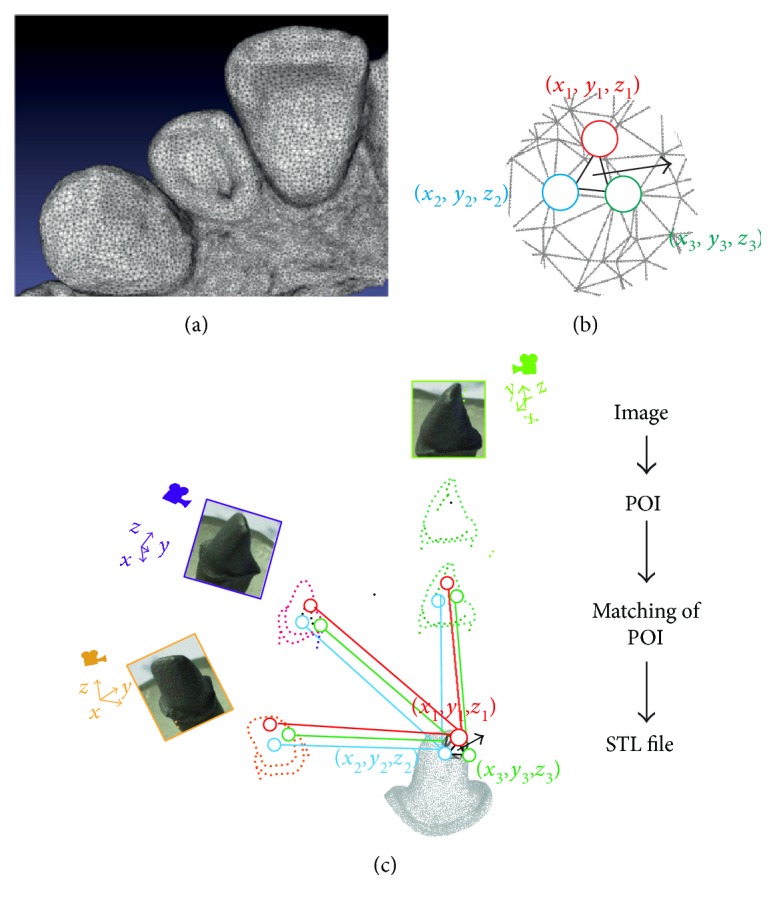
Generation of a STL file by intraoral scanner. (a) An example of a STL file. (b) Each triangle of a STL file is composed by three points with cartesian coordinates (*x*, *y*, and *z*) and a normal surface. (c) Schematic representation of the reconstruction technology: each picture is analyzed, and POI (points of interest) are selected by the software. After similarity calculation between different images, a matching of coinciding POI is defined and triangles with coordinates are generated by projection matrix.

IOS is a medical device composed of a handheld camera (hardware), a computer, and a software. The goal of IOS is to record with precision the three-dimensional geometry of an object. The most widely used digital format is the open STL (Standard Tessellation Language) or locked STL‐like (Figure 1(a)). This format is already used in many industrial fields and describes a succession of triangulated surfaces where each triangle is defined by three points and a normal surface (Figure 1(b)). However, other file formats have been developed to record color, transparency, or texture of dental tissues (such as Polygon File Format, PLY files). Irrespective of the type of imaging technology employed by IOS, all cameras require the projection of light that is then recorded as individual images or video and compiled by the software after recognition of the POI (points of interest). The first two coordinates (*x* and *y*) of each point are evaluated on the image, and the third coordinate (*z*) is then calculated depending on the distance to object technologies of each camera, as explained below (Figure 1(c)).

### 2.1. Light Projection and Capture

**Figure 2:**
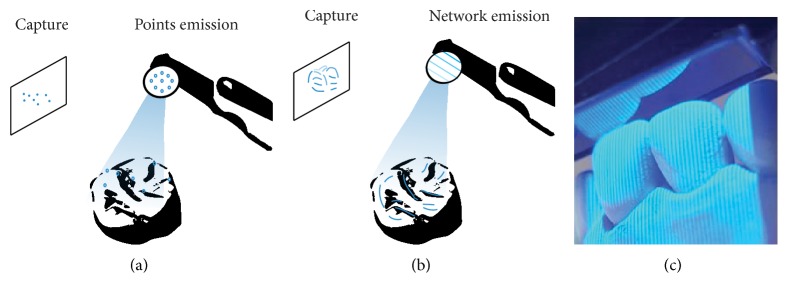
Nature of light. (a) Projection of points. (b) Projection of a mesh. (c) Projection of a mesh by an intraoral scanner.

Within the 3D reconstruction field, there is a clear distinction between passive and active techniques. Passive techniques use only ambient lighting to illuminate intraoral tissues and are reliant on a certain level of texture of an object. Active techniques use white, red, or blue structured lights projected from the camera onto the object that is less reliant on the real texture and color of tissues for reconstruction [8, 9]. In active techniques, a luminous point is projected onto an object and the distance to the object is calculated by triangulation (process explained later) (Figure 2(a)). An alternative is light pattern projection, such as line or mesh projections (Figures 2(b) and 2(c)) [10]. The surface reconstruction can be achieved with a compilation of images, a video that can take several images per second in a continuous data flow, or per wave analysis [11, 12].

### 2.2. Distance to Object Technologies

#### 2.2.1. Triangulation

**Figure 3:**
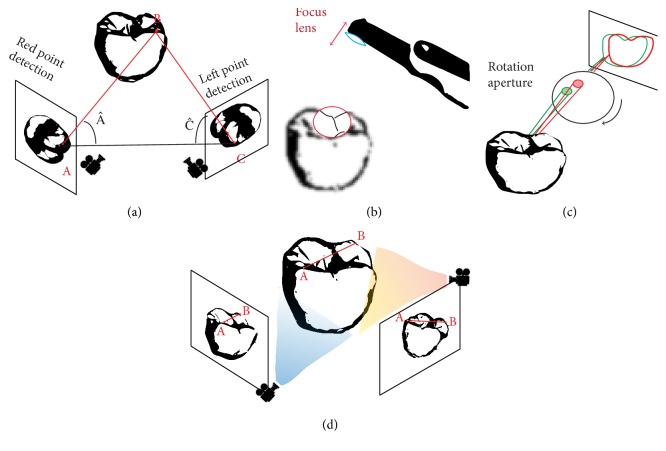
Determining distance to the object. (a) Triangulation: distance BC could be determined according to the formula $BC=AC\times \text{sin}\left(\hat{A}\right)/\text{sin}\left(\hat{A}+\hat{C}\right)$. (b) Confocal: distance to the object is determined according to the focal distance. (c) AWS requiring a camera and an off-axis that moves on a circular path around the optical axis and produces a rotation of interest points. (d) Stereophotogrammetry is a technology that generates files by algorithm analyzing numerous pictures.

Triangulation is based on a principle that the position of a point of a triangle (the object) can be calculated knowing the positions and angles of two points of view (Figure 3(a)). These two points of view may be produced by two detectors, a single detector using a prism, or captured at two different points in time.

#### 2.2.2. Confocal

Confocal imaging is a technique based on acquisition of focused and defocused images from selected depths (Figure 3(b)). This technology can detect the sharpness area of the image to infer distance to the object that is correlated to the focal length of the lens. A tooth can then be reconstructed by successive images taken at different focuses and aperture values and from different angles around the object [12]. The sharpness area is directly related to the dexterity of the operator who can generate motion blur [13], and this technique also requires large optics that may lead to difficulties in clinical practice.

#### 2.2.3. AWS (Active Wavefront Sampling)

AWS is a surface imaging technique, requiring a camera and an off-axis aperture module. The module moves on a circular path around the optical axis and produces a rotation of POI (Figure 3(c)). Distance and depth information are then derived and calculated from the pattern produced by each point [8].

#### 2.2.4. Stereophotogrammetry

Stereophotogrammetry estimates all coordinates (*x*, *y*, and *z*) only through an algorithmic analysis of images [14] (Figure 3(d)). As this approach relies on passive light projection and software rather than active projection and hardware, the camera is relatively small, its handling is easier, and its production is cheaper.

### 2.3. Reconstruction Technologies

One of the major challenges of generating a 3D numerical model is the matching of POI taken under different angles. Distances between different pictures may be calculated using an accelerometer integrated in the camera, but a similarity calculation is more often used to determine the point of view of the image. Using algorithms, similarity calculation defines POI coincident on different images [2]. These POI can be found by detection of transition areas, such as strong curvatures, physical limits, or differences of grey intensity (“Shape from Silhouette”) [15]. A transformation matrix is then calculated to evaluate similarity between all images such as rotation or homothety. Extreme points can also be statistically eliminated to reduce noise. Each coordinate (*x*, *y*, and *z*) is extracted from the projection matrix, and a file is then generated.

## 3. Clinical Impact of IOS Technologies

### 3.1. Handling and Learning

Recent studies have indicated that the digital impression technique was more comfortable and faster than the current impression technique [16–19]. Lee and Gallucci have reported that implant impression with IOS using confocal technology was a more efficient technique with shorter preparation and retake time than conventional implant impressions for inexperienced second year dental students [20]. In two other clinical studies, IOS using confocal or AWS was significantly preferred over conventional impression; it was more time efficient, comfortable, and patient friendly for implant impression [19, 21].

Each scanner also includes specific technology and captors that impact size and weight of the scan head [6]. For instance, technologies such as confocal or AWS are mainly based on hardware that requires voluminous components. However, among the IOS that employ the same technology, clinical differences are reported; it is reported that participants preferred the use of Trios over iTero although these are both based on confocal technology [17]. This is related to the time for operators to familiarize themselves with the ergonomics and software of each IOS, and the learning curve can be initially slow. Indeed, a study compared experience curves between initial scan and after repeated scans using two IOS with confocal technology. It was found that, although scanning time decreased with training for both scanner, the average scanning time for Trios was always shorter than that for iTero [22]. In addition, the software, the technology employed, and the scanning path all seem to affect handling time during digital impression that has been reported to be between 4 and 15 minutes with no clear determining factor [12].

### 3.2. Powdering

**Figure 4:**
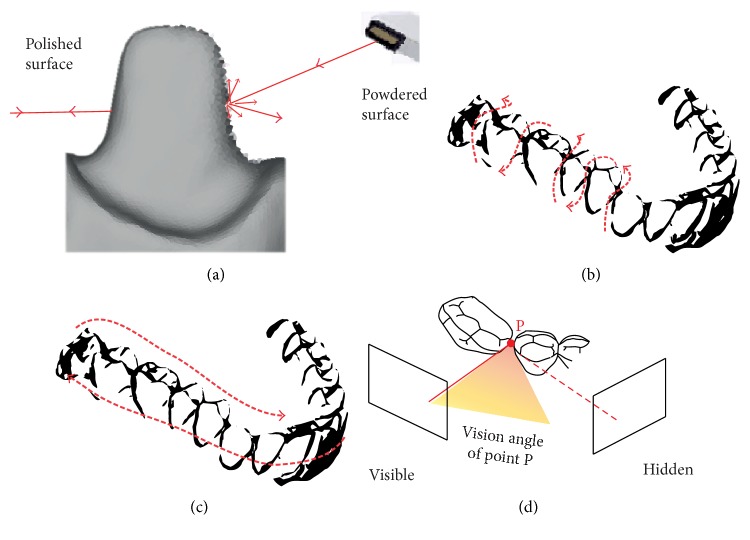
Scanning strategies. (a) Prepared teeth have reflective surfaces due to enamel or polished surface. Powdering can increase diffuse light that diminish this phenomenon. (b) A one-way scan (S sweep on vestibular, occlusal, and lingual surfaces). (c) A linear movement on occlusal-palatal surfaces followed by buccal surface. (d) Proximal faces are hidden if the scanning strategy is not adapted.

Dental tissues present many reflective surfaces, such as enamel crystals or polished surfaces, that could disrupt the matching of POI by the software due to overexposure. To prevent this, practitioners could change the orientation of the camera to increase diffuse light (Figure 4(a)). Another strategy to overcome this difficulty employed by some systems is to use cameras with a polarizing filter [23]. For other scanners, a 20–40 *μ*m powder coating is required during the digitizing process to reduce reflectivity (Figure 4(a)). Theoretically, the powder thickness could vary between operators and reduce file accuracy, but the software of the IOS is capable of taking an average thickness into account [24].

Powder-based digital impression has been previously shown to be very accurate for partial impressions [25, 26]. However, powder could be relatively uncomfortable for patients, and additional scanning time has been reported when powder is contaminated with saliva during impression as this requires cleaning and reapplication of powder [21]. Moreover, concerning full-jaw scans, IOS using powder-free technologies appears to be recommended due to the difficulty to maintain powder coating on all the teeth for the duration of the scan [6]. In conclusion, although powder is not very comfortable for patients, no clear difference was found in articles concerning the effect of powdering on scan accuracy.

### 3.3. Scanning Paths

Scan path means that the intraoral scanner must be used according to a specific movement to increase accuracy of the virtual model [6]. Recent studies have shown the influence of the scan path on the accuracy of data captured using confocal scanners, both in vitro and in vivo [27]. The scanned object should be positioned at the center of an acquisition area to describe an optimal sphere around the object. Practitioners also have to maintain a fluid movement, always preserving a steady distance and the tooth centered during recording. The camera should be held in a range of between 5 and 30 mm of the scanned surface depending on the scanners and technologies [6, 8, 28]. This handling is particularly difficult during the change of axis, such as the passage from posterior to anterior tooth or in case of malposition. Some manufacturers propose guides to avoid practitioners to maintain distance and keep the surrounding tissue out of the field of view of the camera.

For IOS using confocal technology, when a scan of the entire arcade is required, different strategies are described by manufacturers. One is a linear movement on all occlusal-palatal surfaces followed by buccal surface (Figure 4(b)). Another procedure consists of making an S sweep on vestibular, occlusal, and lingual faces of each tooth successively [27, 29] (Figure 4(c)). The first strategy seems to limit spatial distortion by finishing the capture at the initial position, and so avoiding an overall one-way error, but linear or rough movement of vestibular scans could be imprecise on interproximal areas. This technical observation leads practitioners to adapt their clinical protocol in difficult areas such as interproximal zones, tooth preparation, high curvatures of central incisive, and change of axis around canines. However, the capture of areas with a steep downward slope, such as the anterior mandibular area, is often associated with difficulties in the treatment of the image [6]. This limitation underlines the increasing significance of IOS tracking and software that is described below.

### 3.4. Tracking and Software

Sometimes during impression, tracking could be lost which may destabilize the software when distance to the object or scan path is not respected; movement is too fast or too jerky. A scan strategy must be followed beginning, for example, with easy parts (occlusal faces of posterior teeth) so that the software has enough information if tracking is lost. Manufacturers are currently developing different strategies and software algorithms to continue scanning when tracking is lost mainly by recognizing saved geometry of the object. For this, practitioners need to rescan a meaningful area without being stationary to give enough information to the camera and software. The second scan will allow matching the previous POI, and the software will complete this missed area [30]. This rematching of POI is directly influenced by a complex geometry of the object such as high curvatures or many hidden faces that reduce the number of POI and complicates the process for the software [31, 32] (Figure 4(d)).

### 3.5. Mesh Quality

**Figure 5:**
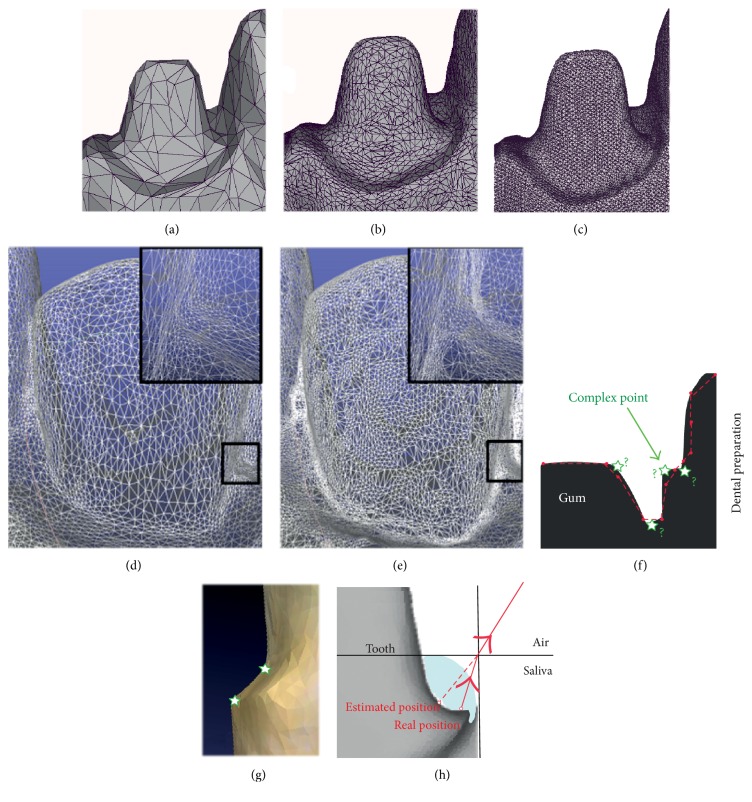
Management of mesh quality. Comparison of STL files depending on mesh density. (a) Low density. (b) Medium density. (c) High density. (d) Large number of triangles over the whole tooth. (e) Routine mesh on flat zones and denser mesh for gingival sulcus. (f) Prepared teeth present various points that are complex to scan. (g) Complex points can appear smoothed on CAD-CAM software. (h) Saliva or water film can generate errors during margin impression that could reduce mesh quality.

The IOS software can generate files of varying mesh densities (Figures 5(a), 5(b), and 5(c)). However, a high mesh density for the whole tooth is not relevant due to high computing time involved. Some files incorporate a routine mesh on flat zones (vestibular face of incisive) and a more dense mesh for high curvatures (incisal edge or gingival sulcus, for example; Figures 5(d) and 5(e)). Indeed, a large number of triangles are sufficient to follow precisely the emergence profile whereas a low number could lead to smoothing of margins (Figures 5(f) and 5(g)). During intraoral scanning, a major difficulty is to control patient mobility that can lead to scanning by mistake peripheral soft tissues such as the tongue or jaws [12]. Similarly, the presence of blood, saliva, or gingival fluid can also falsify the picture acquired [15]. For example, a tight film of water can lead to an error to the order of millimeters on margin impression (Figure 5(h)). The latest IOS also provide color and texture that greatly increase the perception of clinical situations and dental volume.

Nevertheless, the rendering of file in the graphical user interface is often misleading on the accuracy of a scan due to the use of shaders and of smoothing algorithms. A thorough analysis of trueness and precision appears to be more relevant factors to evaluate the scanner accuracy of the current IOS, and these aspects are discussed below.

## 4. Accuracy of IOS Technologies

### 4.1. Definition of IOS Accuracy

According to ISO 5725, the accuracy is described by two measurement methods: trueness and precision [33, 34]. Trueness refers to the closeness of agreement between the arithmetic mean of a large number of test results and the true or accepted reference value. Precision refers to the closeness of agreement between test results. The method of measurement contributes to the variability of trueness and precision reported for IOS, as this depends on aspects such as the operator, equipment used and calibration, the time elapsed between measurements, and the environment (temperature, humidity, etc.). However, the methods to calculate precision and trueness for IOS are limited due to the quality of references used and the measurement technique employed. For instance, in vitro, a plaster model scan using extra oral technology is currently defined as the reference, but it is difficult to compare these results with in vivo files as for the latter a plaster scan obtained from indirect physicochemical impression (i.e., likely to contain inaccuracies) is the reference [34, 35]. Moreover, some studies have compared distances between STL generated from a plaster model and those generated with IOS manually, whereas other studies have used an algorithm to align two different files and calculate the distance between them [36, 37]. However, the process of measurement in the first strategy is highly operator dependent whereas the alignment algorithm requires subjective manual operator suppression of inaccurate areas, such as the tongue or soft tissues, to prevent falsified alignment. Then, further investigations are required to develop standardized and comparable strategies for the measurement of IOS accuracy [34].

### 4.2. Precision and Trueness of IOS Files

Many papers have reported clinically valuable precision and trueness of current IOS, both in vitro and in vivo [16, 38–42]. For example, Ender et al. have reported that the mean trueness of various IOS technologies is between 20 and 48 *μ*m and the precision is between 4 and 16 *μ*m, when the impression is partial and compared to conventional impression [40]. The conclusion of these reports is that current IOS devices are clinically adapted for common practice, with at least similar accuracy to conventional impression taking [6, 41, 42]. However, in vivo full-arch impression is reported to be associated with a phenomenon of distortion, in particular for triangulation, confocal, or AWS technologies [40, 43, 44].

**Figure 6:**
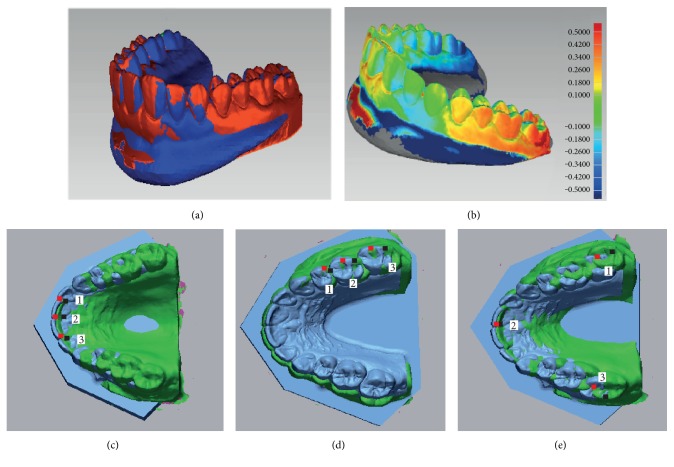
Accuracy of full arch impression and matching process. (a) Full-arch files generated with IOS and laboratory scanner were matched (with Geomagic software). (b) Three-dimensional deviations between IOS and reference files revealed posterior distortion. Impact of selected points (1, 2, and 3) to the matching process (with CloudCompare). (c) Anterior points. (d) Lateral-located points. (e) Scattered points.

Concerning implantology, various in vitro studies concluded that triangulation, confocal and AWS technologies can be feasible alternatives to high-accuracy scans currently used for scanning conventional impressions or plaster models [29, 45–47]. Nevertheless, both in vitro and in vivo studies have reported that distance and angulation errors were currently too large to make multiple implant-based prosthesis (Figures 6(a) and 6(b)), such as for edentulous mandibles, due to the lack of anatomical landmarks for scanning, irrespective of the technology employed. Indeed, compared to teeth, absence of a periodontal ligament limits implant adaptations in case of microscopic error that can lead to implant complications [45, 48, 49].

### 4.3. Intermaxillary Relationship Registration

Dentists always require to take the intermaxillary relationship to perform prosthetic rehabilitation for patients. This complex clinical step is a common source of error due to cumbersome and imprecision of bite registration materials. By contrast, impressions using IOS only require a new acquisition of vestibular faces when the patient is in occlusion [50]. Maxillary and mandibular archs are then aligned with a matching process. Even if this complex algorithm requires coincident areas positioned under different planes (Figures 6(c), 6(d), and 6(e)), a recent study reported that only one left and one right lateral occlusal records are required for software alignment, with a minimum dimension of 12 × 15 mm [50, 51].

## 5. Conclusion

After an objective overview of the literature, IOS seems clinically adapted for common practice, irrespective of the technology used. Each technology has to be considered in the context of individual activity, requirements, and expectations of practitioners. An understanding of the IOS technology is necessary for any practitioner to have a successful clinical strategy during the scanning of prepared teeth. However, there is no scanning technique, scanner, or technology that can currently be unanimously considered more accurate due to the lack of standardized procedures or comparable in vivo studies. Although IOS is currently mainly based on confocal technology, the requirement of voluminous hardware means that alternatives are sought such as software-based technologies, especially for ergonomic reasons, patient comfort, and manufacturing price (20–25 k€ for software based instead of 35–40 k€ for hardware based).

## References

[1] Chen L. C., Xu Z. Q. (2005). Innovative 3D dental measurement for tooth model restoration. *Key Engineering Materials*.

[2] Hong-Seok P., Chintal S. (2015). Development of high speed and high accuracy 3D dental intra oral scanner. *Procedia Engineering*.

[3] Ali A. O. (2015). Accuracy of digital impressions achieved from five different digital impression systems. *Dentistry*.

[4] Duret F. (1985). Toward a new symbolism in the fabrication of prosthetic design. *Les Cahiers de Prothèse*.

[5] Baheti M. J., Soni U. N., Gharat N. V., Mahagaonkar P., Khokhani R., Dash S. (2015). Intra-oral scanners: a new eye in dentistry. *Austin Journal of Orthopedics & Rheumatology*.

[6] Zimmermann M., Mehl A., Mörmann W. H., Reich S. (2015). Intraoral scanning systems - a current overview. *International Journal of Computerized Dentistry*.

[7] Alghazzawi T. F. (2016). Advancements in CAD/CAM technology: options for practical implementation. *Journal of Prosthodontic Research*.

[8] Logozzo S., Zanetti E. M., Franceschini G., Kilpelä A., Mäkynen A. (2014). Recent advances in dental optics - part I: 3D intraoral scanners for restorative dentistry. *Optics and Lasers in Engineering*.

[9] Duret F., Pélissier B. (2010). Différentes méthodes d’empreinte en CFAO dentaire. *EMC (Elsevier Masson SAS, Paris), Médecine Buccale*.

[10] Geng J. (2011). Structured-light 3D surface imaging: a tutorial. *Advances in Optics and Photonics*.

[11] Ireland A. J., McNamara C., Clover M. J. (2008). 3D surface imaging in dentistry - what we are looking at. *British Dental Journal*.

[12] Taneva E., Kusnoto B., Evans C. A. (2015). 3D scanning, imaging, and printing in orthodontics. *Chapter 9 Issues in Contemporary Orthodontics*.

[13] Giménez B., Özcan M., Martínez-Rus F., Pradíes G. (2015). Accuracy of a digital impression system based on active wavefront sampling technology for implants considering operator experience, implant angulation, and depth. *Clinical Implant Dentistry and Related Research*.

[14] Pradíes G., Ferreiroa A., Özcan M., Giménez B., Martínez-Rus F. (2014). Using stereophotogrammetric technology for obtaining intraoral digital impressions of implants. *Journal of the American Dental Association (1939)*.

[15] Aubreton O., Bajard A., Verney B., Truchetet F. (2013). Infrared system for 3D scanning of metallic surfaces. *Machine Vision and Applications*.

[16] Gjelvold B., Chrcanovic B. R., Korduner E. K., Collin-Bagewitz I., Kisch J. (2016). Intraoral digital impression technique compared to conventional impression technique. A randomized clinical trial. *Journal of Prosthodontics*.

[17] Yuzbasioglu E., Kurt H., Turunc R., Bilir H. (2014). Comparison of digital and conventional impression techniques: evaluation of patients’ perception, treatment comfort, effectiveness and clinical outcomes. *BMC Oral Health*.

[18] Patzelt S. B. M., Emmanouilidi A., Stampf S., Strub J. R., Att W. (2014). Accuracy of full-arch scans using intraoral scanners. *Clinical Oral Investigations*.

[19] Burhardt L., Livas C., Kerdijk W., Meer W. J. v. d., Ren Y. (2016). Treatment comfort, time perception, and preference for conventional and digital impression techniques: a comparative study in young patients. *American Journal of Orthodontics and Dentofacial Orthopedics*.

[20] Lee S. J., Gallucci G. O. (2013). Digital vs. conventional implant impressions: efficiency outcomes. *Clinical Oral Implants Research*.

[21] Joda T., Brägger U. (2016). Patient-centered outcomes comparing digital and conventional implant impression procedures: a randomized crossover trial. *Clinical Oral Implants Research*.

[22] Kim J., Park J.-M., Kim M., Heo S.-J., Shin I. H., Kim M. (2016). Comparison of experience curves between two 3-dimensional intraoral scanners. *The Journal of Prosthetic Dentistry*.

[23] Burgner J., Simpson A. L., Fitzpatrick J. M. (2013). A study on the theoretical and practical accuracy of conoscopic holography-based surface measurements: toward image registration in minimally invasive surgery. *International Journal of Medical Robotics and Computer Assisted Surgery*.

[24] da Costa J. B., Pelogia F., Hagedorn B., Ferracane J. L. (2010). Evaluation of different methods of optical impression making on the marginal gap of onlays created with CEREC 3D. *Operative Dentistry*.

[25] Patzelt S. B. M., Lamprinos C., Stampf S., Att W. (2014). The time efficiency of intraoral scanners: an in vitro comparative study. *Journal of the American Dental Association (1939)*.

[26] Hack G. D., Patzelt S. B. M. (2015). Evaluation of the accuracy of six intraoral scanning. *Journal of the American Dental Association (1939)*.

[27] Müller P., Ender A., Joda T., Katsoulis J. (2016). Impact of digital intraoral scan strategies on the impression accuracy using the TRIOS pod scanner. *Quintessence International*.

[28] Logozzo S., Kilpelä A., Mäkynen A., Zanetti E. M., Franceschini G. (2014). Recent advances in dental optics - part II: experimental tests for a new intraoral scanner. *Optics and Lasers in Engineering*.

[29] Van der Meer W. J. V., Andriessen F. S., Wismeijer D., Ren Y. (2012). Application of intra-oral dental scanners in the digital workflow of implantology. *PLoS One*.

[30] Mao Z., Park K., Lee K., Li X. (2014). Robust surface reconstruction of teeth from raw pointsets. *International Journal of Numerical Methods in Biomedical Engineering*.

[31] Yuan T., Liao W., Dai N., Cheng X., Yu Q. (2010). Single-tooth modeling for 3D dental model. *International Journal of Biomedical Imaging*.

[32] Tzou C. H. J., Artner N. M., Pona I. (2014). Comparison of three-dimensional surface-imaging systems. *Journal of Plastic, Reconstructive & Aesthetic Surgery*.

[33] Menditto A., Patriarca M., Magnusson B. (2007). Understanding the meaning of accuracy, trueness and precision. *Accreditation and Quality Assurance*.

[34] Ender A., Mehl A. (2013). Accuracy of complete-arch dental impressions: a new method of measuring trueness and precision. *The Journal of Prosthetic Dentistry*.

[35] Jeong I.-D., Lee J.-J., Jeon J.-H., Kim J.-H., Kim H.-Y., Kim W.-C. (2016). Accuracy of complete-arch model using an intraoral video scanner: an in vitro study. *The Journal of Prosthetic Dentistry*.

[36] Flügge T. V., Att W., Metzger M. C., Nelson K. (2016). Precision of dental implant digitization using intraoral scanners. *The International Journal of Prosthodontics*.

[37] Ender A., Mehl A. (2014). Accuracy in dental medicine, a new way to measure trueness and precision. *Journal of Visualized Experiments*.

[38] Vecsei B., Joós-Kovács G., Borbély J., Hermann P. (2016). Comparison of the accuracy of direct and indirect three-dimensional digitizing processes for CAD/CAM systems – an in vitro study. *Journal of Prosthodontic Research*.

[39] Jacob H. B., Wyatt G. D., Buschang P. H. (2015). Reliability and validity of intraoral and extraoral scanners. *Progress in Orthodontics*.

[40] Ender A., Attin T., Mehl A. (2016). In vivo precision of conventional and digital methods of obtaining complete-arch dental impressions. *The Journal of Prosthetic Dentistry*.

[41] Ahlholm P., Sipilä K., Vallittu P., Jakonen M., Kotiranta U. (2016). Digital versus conventional impressions in fixed prosthodontics: a review. *Journal of Prosthodontics*.

[42] Ting-Shu S., Jian S. (2015). Intraoral digital impression technique: a review. *Journal of Prosthodontics*.

[43] Rhee Y.-K., Huh Y.-H., Cho L.-R., Park C.-J. (2015). Comparison of intraoral scanning and conventional impression techniques using 3-dimensional superimposition. *The Journal of Advanced Prosthodontics*.

[44] Gan N., Xiong Y., Jiao T. (2016). Accuracy of intraoral digital impressions for whole upper jaws, including full dentitions and palatal soft tissues. *PLoS One*.

[45] Giménez B., Özcan M., Martínez-Rus F., Pradíes G. (2015). Accuracy of a digital impression system based on active triangulation technology with blue light for implants: effect of clinically relevant parameters. *Implant Dentistry*.

[46] Giménez B., Pradíes G., Martínez-Rus F., Özcan M. (2015). Accuracy of two digital implant impression systems based on confocal microscopy with variations in customized software and clinical parameters. *The International Journal of Oral & Maxillofacial Implants*.

[47] Gimenez-Gonzalez B., Hassan B., Özcan M., Pradíes G. (2016). An in vitro study of factors influencing the performance of digital intraoral impressions operating on active wavefront sampling technology with multiple implants in the edentulous maxilla. *Journal of Prosthodontics*.

[48] Mangano F. G., Veronesi G., Hauschild U., Mijiritsky E., Mangano C. (2016). Trueness and precision of four intraoral scanners in oral implantology: a comparative in vitro study. *PLoS One*.

[49] Andriessen F. S., Rijkens D. R., Meer W. J. V. D., Wismeijer D. W. (2014). Applicability and accuracy of an intraoral scanner for scanning multiple implants in edentulous mandibles: a pilot study. *The Journal of Prosthetic Dentistry*.

[50] Solaberrieta E., Garmendia A., Brizuela A., Otegi J. R., Pradies G., Szentpétery A. (2016). Intraoral digital impressions for virtual occlusal records: section quantity and dimensions. *BioMed Research International*.

[51] Solaberrieta E., Arias A., Brizuela A., Garikano X., Pradies G. (2016). Determining the requirements, section quantity, and dimension of the virtual occlusal record. *The Journal of Prosthetic Dentistry*.

